# Explicit Solutions of MHD Flow and Heat Transfer of Casson Fluid over an Exponentially Shrinking Sheet with Suction

**DOI:** 10.3390/nano12193289

**Published:** 2022-09-21

**Authors:** Ling Liu, Jing Li, Shijun Liao

**Affiliations:** 1 School of Naval Architecture, Ocean and Civil Engineering, Shanghai Jiao Tong University, Shanghai 200240, China; 2 Marine Numerical Experiment Center, State Key Laboratory of Ocean Engineering, Shanghai 200240, China

**Keywords:** explicit solutions, Casson fluid, heat transfer, MHD flow

## Abstract

In this study, the magnetohydrodynamic (MHD) flow and heat transfer of a Casson fluid over an exponentially shrinking sheet with suction is investigated using the homotopy analysis method (HAM). Different from previous numerical methods and analytical techniques, we have obtained an explicit formula solution to the presented nonlinear problem. The explicit solutions of f(η) and θ(η) are obtained and are valid in the whole domain. The changes in velocity and temperature profiles are studied in cases of different Casson fluid parameter γ, magnetic interaction parameter *M*, suction parameter *s*, and Prandtl number Pr. The convergent solutions are verified by comparison with the numerical results. In addition, the skin friction coefficient $C_{f}$ and local Nusselt number $Nu_{x}$ are analyzed using the analytic formulas of $f^{\prime \prime }\left(0\right)$ and $\theta^{\prime }\left(0\right)$, respectively. The analytical formulas help us intuitively analyze the influence of various parameters at the theoretical level. The effects of different physical quantities on $C_{f}$ and $Nu_{x}$ are thoroughly investigated.

## 1. Introduction

Magnetohydrodynamics (MHD) refers to the study of the properties of magnetic fields and electrically conducting fluids. It focuses on the mutual interaction between fluid flow and magnetic fields. Thus, the fluids are required to be electrically conductive and non-magnetic, e.g., saltwater, plasmas, liquid metals, and electrolytes. MHD flows have found application in many branches of fluid mechanics [1,2,3,4]. The appearance of three-dimensional objects in wall-bounded MHD flows are characterized experimentally [5]. Reddy et al. [6] obtained numerical solutions of MHD flows in an electrically conductive fluid driven by a traveling magnetic field imposed at the end caps of a cylindrical annulus. Camobreco et al. [7] examined base flow influence in the context of the transition to turbulence in a quasi-two-dimensional MHD flow. With the development of MHD flow, many researchers have begun paying attention to boundary layer problems in MHD flow.

Most boundary layer problems considered in MHD flow are nonlinear. The flows are usually governed by one- or multiple-coupled nonlinear ordinary differential equations (ODEs). Thus, it is of great significance to develop effective methods to solve these nonlinear problems. Many researchers have conducted investigations on MHD boundary layer flow. Mukhopadhyay et al. [8] numerically studied the MHD boundary layer flow over a heated stretching sheet with variable viscosity. We investigate an MHD free-convection boundary layer flow saturated in a Darcian–Forchheimer porous medium over a vertical flat plate in the presence of suction/injection effect. This is conducted numerically by employing the routine bvp4c of the symbolic computer algebra software MATLAB [9]. The MHD boundary layer flow over a nonlinear stretching sheet is studied by a direct collocation method based on rational Legendre functions [10]. In addition to the MHD boundary layer problem of Newtonian fluids, attention has also increasingly shifted toward the certain flow problems of non-Newtonian fluids acting on the MHD boundary layer.

Recently, MHD boundary layer flow combined with Casson fluid has attracted the interest of many researchers. As a non-Newtonian fluid, Casson fluid is very common in nature and man-made products, such as jelly, soap, honey, and human blood. Some theoretical analyses of the related MHD Casson fluid flow have been conducted by several researchers through various numerical techniques [11,12,13,14]. Although there exists an abundance of numerical methods to deal with the nonlinear MHD boundary layer flow of a Casson fluid, the obtainment of an explicit analytical approximation of these nonlinear problems remains a challenging task.

We note that Nadeema et al. [15] employed the Adomian Decomposition Method (ADM) and numerically studied the MHD boundary layer flow of a Casson fluid induced by an exponentially shrinking sheet. In their work, the heat transfer of Casson fluid over the exponentially shrinking sheet is not considered. However, heat transfer is a crucial aspect applied in different engineering areas such as materials, energy, machinery, chemical industry, medicine, and other fields. Casson fluid is a typical non-Newtonian fluid, and it is of great significance to study its heat transfer characteristics in MHD boundary layer flow in practice. To the best of our knowledge, no explicit analytical solutions have been presented for the MHD flow and heat transfer of a Casson fluid over an exponentially shrinking sheet. This provides the inspiration behind us conducting this study.

In this paper, we apply the homotopy analysis method (HAM) [16,17,18,19] to the nonlinear MHD flow and heat transfer of a Casson fluid over an exponentially shrinking sheet with suction. The HAM method allows great freedom in the selection of proper base functions, auxiliary linear operators, initial guesses of unknowns, and convergence-control parameters. This method provides a straightforward way to ensure the convergence of solution series in nonlinear problems even for strongly nonlinear equations. Nowadays, it is widely applied to solve many strongly nonlinear problems in different areas [20,21,22,23,24,25,26,27]. The success of these applications demonstrates the great potential of the HAM method. In particular, the HAM method has been used to deal with many boundary layer flows, such as Blasius’ viscous flow [28], MHD Falkner–Skan flow of nano-fluids [29], Casson fluid flow with stretching sheet [30], and fluid flow over an exponentially stretching porous sheet [31]. These encourage us to employ the HAM method to the present boundary layer flow and further improve it.

We emphasize that explicit analytic formulas for the velocity and temperature distribution in a considered system do not currently exist, and which should also be valid in the whole domain. This motivates us to solve the problem. In the next section, we provide the mathematical model and formulas of the present boundary layer flow. The explicit analytic solutions of the current system are given in Section 3. In Section 4, we test the obtained explicit analytic solutions. The convergence and accuracy of solutions are evaluated in detail. Discussions concerning the effects of various physical parameters are investigated in Section 5. Summaries are presented in the last Section 6.

## 2. Mathematical Model

**Figure 1:**
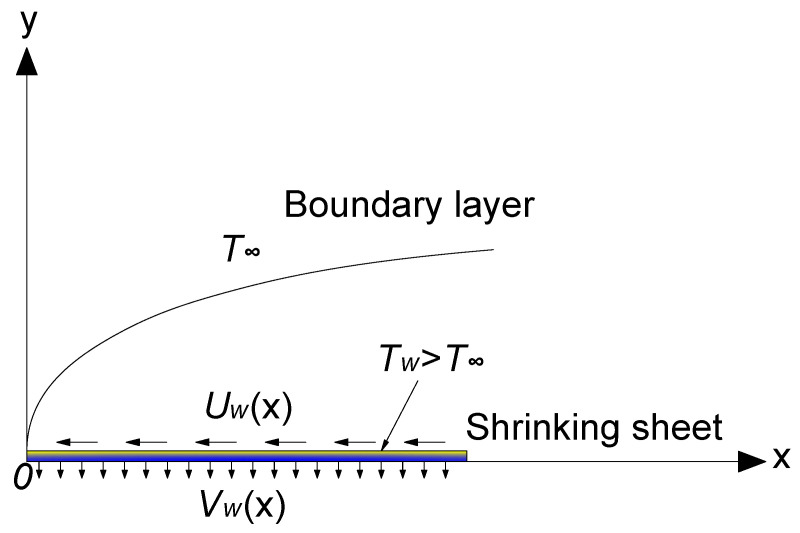
Physical model and coordinate system.

Consider the steady two-dimensional MHD boundary layer flow of a Casson fluid over an exponentially shrinking sheet. As illustrated in Figure 1, we assume that $U_{w}\left(x\right)$ denotes the velocity of the shrinking sheet, $V_{m}\left(x\right)$ is the variable wall mass transfer velocity, $T_{w}$ is the uniformly distributed sheet temperature, $T_{\infty }$ is the free stream temperature assumed to be constant, where

$T_{w}>T_{\infty }$ corresponds to the heated shrinking sheet. The fluid is electrically conductive with the uniform magnetic field applied normal to the shrinking sheet. Under the boundary layer approximations, the governing equations of continuity, motion, and energy equations are written as

(1) $$\begin{array}{cc} \; & \frac{\partial u}{\partial x}+\frac{\partial v}{\partial y}=0, \end{array}$$

(2) $$\begin{array}{cc} \; & u\frac{\partial u}{\partial x}+v\frac{\partial u}{\partial y}=\nu \left(1+\frac{1}{\gamma }\right)\frac{\partial^{2}u}{\partial y^{2}}-\frac{\sigma B^{2}}{\rho }u, \end{array}$$

(3) $$\begin{array}{cc} \; & u\frac{\partial T}{\partial x}+v\frac{\partial T}{\partial y}=\alpha \frac{\partial^{2}T}{\partial y^{2}}, \end{array}$$

where *u* and *v* are the corresponding velocities in *x*- and *y*-directions, respectively. ν=μ/ρ is the kinematic fluid viscosity, ρ is the fluid density, and μ is the Casson viscosity coefficient. γ is the Casson fluid parameter. σ is the electrical conductivity of the Casson fluid. $B=B_{0}exp\left(x/L\right)$ denotes the magnetic field, where $B_{0}$ is the constant magnetic field, and *L* is the characteristic length. $\alpha =\kappa /(\rho c_{p})$ is the thermal diffusivity, where κ is the fluid thermal conductivity and $c_{p}$ is the specific heat. The boundary conditions of governing Equations (1)–(3) are given by

(4) $$\begin{array}{cc} \; & u=U_{w}=-U_{0}\;exp\left(\frac{x}{L}\right),\;\mathrm{at}\;y=0, \end{array}$$

(5) $$\begin{array}{cc} \; & v=V_{w}=V_{0}\;exp\left(\frac{x}{2L}\right),\;\mathrm{at}\;y=0, \end{array}$$

(6) $$\begin{array}{cc} \; & T=T_{w}=T_{\infty }+T_{0},\;\mathrm{at}\;y=0, \end{array}$$

(7) $$\begin{array}{cc} \; & U=0,\;T=T_{\infty },\;\mathrm{as}\;y\to \infty \end{array}$$

where $U_{0}>0$ is shrinking constant, $V_{0}$ is a constant with $V_{0}<0$ for masss suction, $T_{0}$ is a constant measuring the uniform increase in temperature along the shrinking sheet.

The governing Equations (1)–(3) are nonlinear partial differential equations. In order to obtain the self-similar equations, we use the similarity transformation reported by Refs. [15,32], and thus Equations (1)–(3) are reduced to the nonlinear ordinary differential equations

(8) $$\begin{array}{cc} \; & \left(1+1/\gamma \right)f^{\prime \prime \prime }\left(\eta \right)-Mf^{\prime }\left(\eta \right)+f\left(\eta \right)f^{\prime \prime }\left(\eta \right)-2f^{\prime 2}\left(\eta \right)=0, \end{array}$$

(9) $$\begin{array}{cc} \; & \theta^{\prime \prime }\left(\eta \right)+Prf\left(\eta \right)\theta^{\prime }\left(\eta \right)=0, \end{array}$$

with boundary conditions

(10) $$\begin{array}{c} f\left(0\right)=s,\;f^{\prime }\left(0\right)=-1,\;f^{\prime }\left(+\infty \right)=0,\;\theta \left(0\right)=1,\;\theta \left(+\infty \right)=0, \end{array}$$

where the prime denotes the differentiation with respect to η, given by $\eta =y\sqrt{\frac{U_{0}}{2\nu L}}e^{\frac{x}{2L}}$, f(η) is related to the stream function ψ by $f\left(\eta \right)=\psi /\left(\sqrt{2\nu LU_{0}}e^{x/2L}\right)$, $\theta \left(\eta \right)=\left(T-T_{\infty }\right)/\left(T_{w}-T_{\infty }\right)$, $M=2L\sigma B^{2}/\rho U_{0}$ is the magnetic interaction parameter, $Pr=\mu c_{p}/\kappa$ is the Prandtl number, and $s=-V_{0}\sqrt{2L/\nu U_{0}}$ is the suction parameter.

The related skin friction coefficient $C_{f}$ and local Nusselt number $Nu_{x}$ are given by using the similarity variables

(11) $$\begin{array}{c} \sqrt{Re_{x}}C_{f}=\left(1+1/\gamma \right)f^{\prime \prime }\left(0\right), \end{array}$$

(12) $$\begin{array}{c} \frac{Nu_{x}}{\sqrt{Re_{x}}}=-\theta^{\prime }\left(0\right), \end{array}$$

where $Re_{x}$ is the local Reynolds number.

## 3. Explicit Solutions

For the current problem, considering the convenience of calculation and obtaining the convergent solution [28], we first introduce a spatial scale factor λ=3 to transform Equations (8) and (9) into

(13) $$\begin{array}{cc} \; & \left(1+\frac{1}{\gamma }\right)\lambda^{2}F^{\prime \prime \prime }\left(\xi \right)-MF^{\prime }\left(\xi \right)+\lambda F\left(\xi \right)F^{\prime \prime }\left(\xi \right)-2\lambda F^{\prime 2}\left(\xi \right)=0, \end{array}$$

(14) $$\begin{array}{cc} \; & \Theta^{\prime \prime }\left(\xi \right)+PrF\left(\xi \right)\Theta^{\prime }\left(\xi \right)=0, \end{array}$$

by the transformation ξ=λη, F(ξ)=f(η), and Θ(ξ)=θ(η). The corresponding boundary conditions (10) become

(15) $$\begin{array}{c} F\left(0\right)=s,\;F^{\prime }\left(0\right)=-\frac{1}{\lambda },\;F^{\prime }\left(+\infty \right)=0,\;\Theta \left(0\right)=1,\;\Theta \left(+\infty \right)=0. \end{array}$$

In the framework of HAM, a nonlinear problem is transformed into an infinite number of linear sub-problems. According to Equations (13)–(15), F(ξ) and Θ(ξ) should hold the following “solution expressions” form

(16) $$\begin{array}{cc} F(\xi ) & =\sum_{m=0}^{+\infty }b_{m,0}^{0}+\sum_{m=0}^{+\infty }\sum_{n=1}^{+\infty }\sum_{k=0}^{+\infty }\;b_{m,n}^{k}\;\xi^{k}exp\left(-n\xi \right), \end{array}$$

(17) $$\begin{array}{cc} \Theta (\xi ) & =\sum_{m=0}^{+\infty }\sum_{n=1}^{+\infty }\sum_{l=0}^{+\infty }\;d_{m,n}^{l}\;\xi^{l}exp\left(-n\xi \right), \end{array}$$

where $b_{m,n}^{k}$ and $d_{m,n}^{l}$ are constant coefficients to be determined by HAM. The solution expressions (16)–(17) guide us to choose the following auxiliary linear operator

(18) $$\begin{array}{c} {£}_{F}\left[\Phi \left(\xi ;q\right)\right]=\frac{d^{3}\Phi }{d\xi^{3}}+\frac{d^{2}\Phi }{d\xi^{2}}, \end{array}$$

(19) $$\begin{array}{c} {£}_{\Theta }\left[\Psi \left(\xi ;q\right)\right]=\frac{d^{2}\Psi }{d\xi^{2}}+\frac{d\Psi }{d\xi }, \end{array}$$

with the properties

(20) $$\begin{array}{cc} \; & {£}_{F}\left[C_{1}\xi +C_{2}exp\left(-\xi \right)+C_{3}\right]=0, \end{array}$$

(21) $$\begin{array}{cc} \; & {£}_{\Theta }\left[C_{4}+C_{5}exp\left(-\xi \right)\right]=0, \end{array}$$

where $C_{1}$, $C_{2}$, $C_{3}$, $C_{4}$ and $C_{5}$ are constants. Considering Equations (15)–(17), the following initial guesses are chosen

(22) $$\begin{array}{ccc} F_{0}\left(\xi \right) & = & s-\frac{1}{\lambda }+\frac{1}{\lambda }e^{-\xi }, \end{array}$$

(23) $$\begin{array}{ccc} \Theta_{0}\left(\xi \right) & = & e^{-\xi }. \end{array}$$

Then, according to Equations (13) and (14), we construct a family of zeroth-order deformation equations

(24) $$\begin{array}{l} \left(1-q\right){£}_{F}\left[\Phi \left(\xi ;q\right)-F_{0}\left(\xi \right)\right]\\ =q\;c_{0}^{f}\left\{\left(1+\frac{1}{\gamma }\right)\lambda^{2}\frac{\partial^{3}\Phi \left(\xi ;q\right)}{\partial \xi^{3}}-M\frac{\partial \Phi (\xi ;q)}{\partial \xi }+\lambda \Phi \left(\xi ;q\right)\frac{\partial^{2}\Phi \left(\xi ;q\right)}{\partial \xi^{2}}-2\lambda {\left[\frac{\partial^{2}\Phi \left(\xi ;q\right)}{\partial \xi^{2}}\right]}^{2}\right\}, \end{array}$$

(25) $$\begin{array}{cc} \; & \left(1-q\right){£}_{\Theta }\left[\Psi \left(\xi ;q\right)-\Theta_{0}\left(\xi \right)\right]=q\;c_{0}^{\theta }\left\{\lambda \frac{\partial^{2}\Psi \left(\xi ;q\right)}{\partial \xi^{2}}+Pr\Phi \left(\xi ;q\right)\frac{\partial \Psi (\xi ;q)}{\partial \xi }\right\}, \end{array}$$

with boundary conditions

(26) $$\begin{array}{cc} \; & \Phi \left(0;q\right)=s,\;\Phi^{\prime }\left(0;q\right)=-\frac{1}{\lambda },\;\Phi^{\prime }\left(+\infty ;q\right)=0,\\ & \Psi (0;q)=1,\;\Psi (+\infty ;q)=0, \end{array}$$

where q∈[0,1] is an embedding parameter, and $c_{0}^{f}$ and $c_{0}^{\theta }$ are two non-zero convergence-control parameters. When q=0, we have the solution

(27) $$\Phi \left(\xi ;0\right)=F_{0}\left(\xi \right),\Psi \left(\xi ;0\right)=\Theta_{0}\left(\xi \right),$$

and at q=1, Equations (24)–(26) are the same as Equations (13)–(15), respectively, so that

(28) Φ(ξ;1)=F(ξ),Ψ(ξ;1)=Θ(ξ).

It is clear that the embedding parameter *q* plays the role of mapping. In other words, when *q* gradually increases from 0 to 1, this mapping ensures Φ(ξ;q) and Ψ(ξ;q) continuously deform from the initial guesses $F_{0}\left(\xi \right)$ and $\Theta_{0}\left(\xi \right)$ to the exact solutions F(ξ) and Θ(ξ), respectively.

For this direct mapping, we express Φ(ξ;q) and Ψ(ξ;q) in Maclaurin series

(29) $$\Phi \left(\xi ;q\right)=\Phi \left(\xi ;0\right)+\sum_{m=1}^{+\infty }\frac{q^{m}}{m!}\frac{\partial^{m}\Phi \left(\xi ;q\right)}{\partial q^{m}}{|}_{q=0}=F_{0}\left(\xi \right)+\sum_{m=1}^{+\infty }F_{m}\left(\xi \right)q^{m},$$

(30) $$\Psi \left(\xi ;q\right)=\Psi \left(\xi ;0\right)+\sum_{m=1}^{+\infty }\frac{q^{m}}{m!}\frac{\partial^{m}\Psi \left(\xi ;q\right)}{\partial q^{m}}{|}_{q=0}=\Theta_{0}\left(\xi \right)+\sum_{m=1}^{+\infty }\Theta_{m}\left(\xi \right)q^{m},$$

respectively. Note that the convergence of the above series is related to the two auxiliary linear operators ${£}_{F}$, ${£}_{\Theta }$, and convergence-control parameters $c_{0}^{f}$, $c_{0}^{\theta }$. Fortunately, the HAM method allows us to choose them freely. This point is different from other analytic methods, due to the absence of physical meaning regarding the convergence-control parameter. If all of them are properly selected, we obtain via Equation (28) that

(31) $$F\left(\xi \right)=F_{0}\left(\xi \right)+\sum_{m=1}^{+\infty }F_{m}\left(\xi \right)\approx F_{0}\left(\xi \right)+\sum_{m=1}^{\bar{M}}F_{m}\left(\xi \right),$$

(32) $$\Theta \left(\xi \right)=\Theta_{0}\left(\xi \right)+\sum_{m=1}^{+\infty }\Theta_{m}\left(\xi \right)\approx \Theta_{0}\left(\xi \right)+\sum_{m=1}^{\bar{M}}\Theta_{m}\left(\xi \right),$$

where $\bar{M}$ is a sufficiently large truncation number and denotes $\bar{M}$th-order approximation. $F_{m}\left(\xi \right)$ and $\Theta_{m}\left(\xi \right)$ (m≥1) are calculated from the corresponding governing equations

(33) $$\begin{array}{cc} \; & {£}_{F}\left[F_{m}\left(\xi \right)-\chi_{n}F_{m-1}\left(\xi \right)\right]\\ \; & =c_{0}^{f}\left\{\left(1+\frac{1}{\lambda }\right)\lambda^{2}F_{m-1}^{\prime \prime \prime }\left(\xi \right)-MF_{m-1}^{\prime }\left(\xi \right)+\sum_{i=0}^{m-1}F_{i}^{\prime \prime }\left(\xi \right)F_{m-1-i}\left(\xi \right)-2\lambda \sum_{i=0}^{m-1}F_{i}^{\prime }\left(\xi \right)F_{m-1-i}^{\prime }\left(\xi \right)\right\}\\ \; & =c_{0}^{f}\;R_{m-1}^{F}\left(\xi \right), \end{array}$$

(34) $$\begin{array}{cc} \; & {£}_{\Theta }\left[\Theta_{m}\left(\xi \right)-\chi_{m}\Theta_{m-1}\left(\xi \right)\right]=c_{0}^{\theta }\left\{\lambda \Theta_{m-1}^{\prime \prime }\left(\xi \right)+Pr\sum_{i=0}^{m-1}F_{m-1-i}\left(\xi \right)\Theta_{i}^{\prime }\left(\xi \right)\right\}=c_{0}^{\theta }\;R_{m-1}^{\Theta }\left(\xi \right), \end{array}$$

with the boundary conditions

(35) $$\begin{array}{c} F_{m}\left(0\right)=F_{m}^{\prime }\left(0\right)=F_{m}^{\prime }\left(+\infty \right)=0,\;\;\Theta_{m}\left(0\right)=\Theta_{m}\left(+\infty \right)=0,\; \end{array}$$

where the right-hand terms $R_{m-1}^{F}\left(\xi \right)$ and $R_{m-1}^{\Theta }\left(\xi \right)$ are derived by substituting Equations (29) and (30) into Equations (24)–(26) and equalizing the like-powers of *q*. $\chi_{m}$ is defined by

(36) $$\chi_{m}=\left\{\begin{array}{cc} 0, & \;\mathrm{when}\;m\leq 1,\\ 1, & \;\mathrm{otherwise}. \end{array}\right.$$

It should be emphasized that Equations (33) and (34) are linear. Thus, first considering the initial guesses (22)–(23), then $F_{1}\left(\xi \right)$, $F_{2}\left(\xi \right)$, *…*, $F_{m}\left(\xi \right)$ and $\Theta_{1}\left(\xi \right)$, $\Theta_{2}\left(\xi \right)$, *…*, $\Theta_{m}\left(\xi \right)$ can be calculated by solving these linear equations. Interestingly, we can now find the structures of $F_{m}\left(\xi \right)$ and $\Theta_{m}\left(\xi \right)$ under each *m*th-order approximation. Then the recurrence formulas regarding the coefficients $b_{m,n}^{k}$ of Equation (16) and $d_{m,n}^{l}$ of Equation (17) are deduced through a lengthy derivation process. For details, please refer to the process in [28]. Here, we list the explicit recurrence formulas of $b_{m,n}^{k}$ in Equation (16) and $d_{m,n}^{l}$ in Equation (17).

### 3.1. $b_{m,n}^{k}$ and f(η)

The coefficients $b_{m,n}^{k}$ in Equation (16) are as follows:

(37) $$b_{0,0}^{0}=s-\frac{1}{\lambda },\;b_{0,1}^{0}=\frac{1}{\lambda },$$

and for m≥1, 0≤n≤m+1 and 0≤k≤m−n+1,

(38) $$\begin{array}{cc} b_{m,0}^{0}= & \chi_{m}\;b_{m-1,0}^{0}-\sum_{q=0}^{m-1}\Gamma_{m,1}^{q}\mu_{1,1}^{q}-\sum_{n=2}^{m+1}\left(n-1\right)\Gamma_{m,n}^{0}\mu_{n,0}^{0}-\sum_{n=2}^{m}\sum_{q=1}^{m-n+1}\Gamma_{m,n}^{q}\left(\left(n-1\right)\mu_{n,0}^{q}-\mu_{n,1}^{q}\right), \end{array}$$

(39) $$b_{m,0}^{1}=0,$$

(40) $$\begin{array}{cc} b_{m,1}^{0}= & \chi_{m}b_{m-1,1}^{0}+\sum_{q=0}^{m-1}\Gamma_{m,1}^{q}\mu_{1,1}^{q}+\sum_{n=2}^{m+1}n\Gamma_{m,n}^{0}\mu_{n,0}^{0}+\sum_{n=2}^{m}\sum_{q=1}^{m-n+1}\Gamma_{m,n}^{q}\left(n\mu_{n,0}^{q}-\mu_{n,1}^{q}\right), \end{array}$$

(41) $$b_{m,1}^{k}=\chi_{m}\;b_{m-1,1}^{k}+\sum_{q=k-1}^{m-1}\Gamma_{m,1}^{q}\mu_{1,k}^{q},\;1\leq k\leq m-1,$$

(42) $$b_{m,1}^{m}=\Gamma_{m,1}^{m-1}\mu_{1,m}^{m-1},$$

(43) $$\begin{array}{cc} \; & b_{m,n}^{k}=\chi_{m}\;b_{m-1,n}^{k}-\sum_{q=k}^{m-n+1}\Gamma_{m,n}^{q}\mu_{n,k}^{q},\;0\leq k\leq m-n,\;2\leq n\leq m, \end{array}$$

(44) $$\begin{array}{cc} \; & b_{m,n}^{m-n+1}=-\Gamma_{m,n}^{m-n+1}\mu_{n,m-n+1}^{m-n+1},\;2\leq n\leq m, \end{array}$$

(45) $$b_{m,m+1}^{0}=-\Gamma_{m,m+1}^{0}\mu_{m+1,0}^{0},$$

for m≥1, please refer to Appendix A for $\Gamma_{m,n}^{q}$ and $\mu_{n,k}^{q}$ mentioned in above formulas.

Therefore, owing to the transformation ξ=λη, f(η)=F(ξ), we obtain the explicit analytic solution of f(η)

(46) $$\begin{array}{cc} f(\eta )= & \lim_{\bar{M}\to +\infty }\sum_{m=0}^{\bar{M}}f_{m}\left(\eta \right)\\ = & \lim_{\bar{M}\to +\infty }\sum_{m=0}^{\bar{M}}b_{m,0}^{0}+\lim_{\bar{M}\to +\infty }\sum_{n=1}^{\bar{M}+1}exp\left(-n\lambda \eta \right)\times \left(\sum_{m=n-1}^{\bar{M}}\sum_{k=0}^{m-n+1}b_{m,n}^{k}{\left(\lambda \eta \right)}^{k}\right), \end{array}$$

### 3.2. $d_{m,n}^{l}$ and θ(η)

The coefficients $d_{m,n}^{l}$ in Equation (17) are as follows:

(47) $$\begin{array}{cc} \; & d_{0,1}^{0}=1,\;d_{m,0}^{0}=0,\;m\geq 1, \end{array}$$

(48) $$\begin{array}{cc} \; & d_{m,1}^{0}=\chi_{m}d_{m-1,1}^{0}-\sum_{n=2}^{m+1}\sum_{q=0}^{m-n+1}\Omega_{m,n}^{q}w_{n,0}^{q}, \end{array}$$

(49) $$\begin{array}{cc} \; & d_{m,1}^{l}=\chi_{m}d_{m-1,1}^{l}+\sum_{q=l}^{m}\Omega_{m,1}^{q-1}w_{1,l}^{q-1},\;1\leq l\leq m-1, \end{array}$$

(50) $$\begin{array}{cc} \; & d_{m,1}^{m}=\Omega_{m,1}^{m-1}w_{1,m}^{m-1}, \end{array}$$

(51) $$\begin{array}{cc} \; & d_{m,n}^{l}=\chi_{m}d_{m-1,n}^{l}+\sum_{q=l}^{m-n+1}\Omega_{m,n}^{q}w_{n,l}^{q},\;2\leq n\leq m,\;0\leq l\leq m-n, \end{array}$$

(52) $$\begin{array}{cc} \; & d_{m,n}^{l}=\sum_{q=l}^{m-n+1}\Omega_{m,n}^{q}w_{n,l}^{q},\;2\leq n\leq m,\;l=m-n+1, \end{array}$$

(53) $$\begin{array}{cc} \; & d_{m,m+1}^{0}=\Omega_{m,m+1}^{0}w_{m+1,0}^{0}, \end{array}$$

for m≥1, the details of $\Omega_{m,n}^{q}$ and $w_{n,l}^{q}$ are listed in Appendix B.

Similarly, the explicit expression of θ(η) is gained

(54) $$\begin{array}{c} \theta \left(\eta \right)=\lim_{\bar{M}\to +\infty }\sum_{m=1}^{\bar{M}}\theta_{m}\left(\eta \right)=\lim_{\bar{M}\to +\infty }\sum_{n=1}^{\bar{M}+1}exp\left(-n\lambda \eta \right)\left(\sum_{m=n-1}^{\bar{M}}\sum_{l=0}^{m-n+1}d_{m,n}^{l}{\left(\lambda \eta \right)}^{l}\right). \end{array}$$

Using all of the above recurrence formulas, the coefficients $b_{m,n}^{k}$ and $d_{m,n}^{k}$ are easily calculated in turn. It is worth emphasizing that the series solutions (46) and (54) are convergent as long as the convergence-control parameters are properly selected. Thus, one can gain accurate results under different values of γ, *M*, and *s*.

In particular, the total averaged value of the squared residual error in the governing equations [19] is evaluated by substituting the $\bar{M}$th-order approximations (46) and (54) into the original governing Equations (8) and (9), respectively. The squared residual error clearly indicates the accuracy of the analytic approximations (46)–(54). It is crucial to guarantee the convergence of an approximation series. As Liao [19] reported, the series approximations contain the convergence-control parameters $c_{0}^{f}$ and $c_{0}^{\theta }$. Thus, the squared residual errors also contain $c_{0}^{f}$ and $c_{0}^{\theta }$. The proper values of $c_{0}^{f}$ and $c_{0}^{\theta }$ can always be found to guarantee the convergence of the homotopy series owing to the great freedom. Obviously, at the given order of approximation $\bar{M}$, the optimal approximation is defined by minimizing the squared residual error with the corresponding optimal convergence-control parameters $c_{0}^{f^{*}}$ and $c_{0}^{\theta^{*}}$, respectively.

## 4. Convergence Test

Physically, the values of $f^{\prime \prime }\left(0\right)$ and $\theta^{\prime }\left(0\right)$ are of great significance. They are related to the skin friction coefficient $C_{f}$ (11) and the local Nusselt number $Nu_{x}$ (12), respectively. In this section, we first give the analytical formulas of $f^{\prime \prime }\left(0\right)$ and $\theta^{\prime }\left(0\right)$ and then test their convergence.

Through the use of Equations (46) and (54), the $\bar{M}$th-order approximation of $f^{\prime \prime }\left(0\right)$ and $\theta^{\prime }\left(0\right)$ are derived

(55) $$\begin{array}{cc} \; & f^{\prime \prime }\left(0\right)=\sum_{n=1}^{\bar{M}+1}\sum_{m=n-1}^{\bar{M}}\lambda^{2}\left(n^{2}b_{m,n}^{0}-2n\beta_{m,n}^{1}b_{m,n}^{1}+2\beta_{m,n}^{2}b_{m.n}^{2}\right), \end{array}$$

(56) $$\begin{array}{cc} \; & \theta^{\prime }\left(0\right)=\sum_{n=1}^{\bar{M}+1}\sum_{m=n-1}^{\bar{M}}\lambda \left(\Lambda_{m,n}^{1}d_{m,n}^{1}-nd_{m.n}^{0}\right), \end{array}$$

where $\beta_{m,n}^{k}$ is defined by Equation (A14), and $\Lambda_{m,n}^{l}$ reads

(57) $$\Lambda_{m,n}^{l}=\left\{\begin{array}{cc} 0, & \;m=n=0,\;l\geq 0,\\ 0, & \;m>0,\;n=0,\;l\geq 0,\\ 0, & \;n>m+1,\\ 0, & \;l>m-n+1\\ 1, & \;\mathrm{otherwise}, \end{array}\right.$$

**Figure 2:**
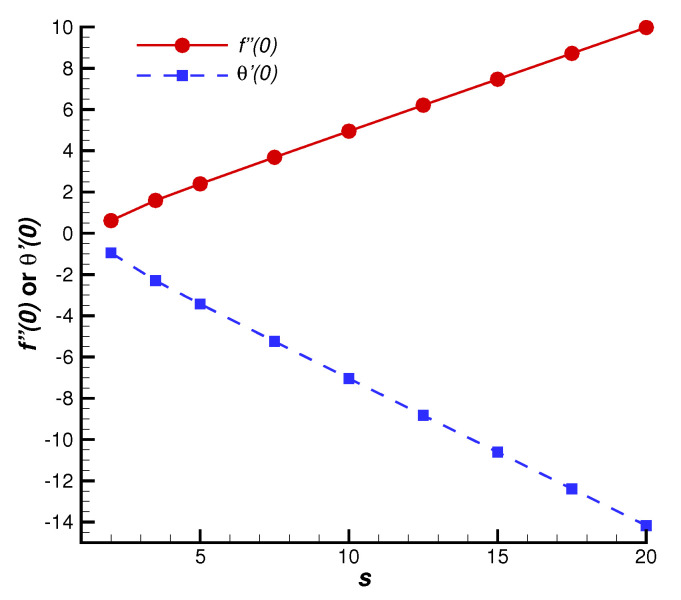
Comparison of convergent series solutions of $f^{\prime \prime }\left(0\right)$ and $\theta^{\prime }\left(0\right)$ obtained by HAM with the numerical results for different values of suction parameter *s* when γ=M=1, Pr=0.71. Solid line: convergent series solutions of $f^{\prime \prime }\left(0\right)$; dashed line: convergent series solutions of $\theta^{\prime }\left(0\right)$; circles: corresponding numerical results.

**Figure 3:**
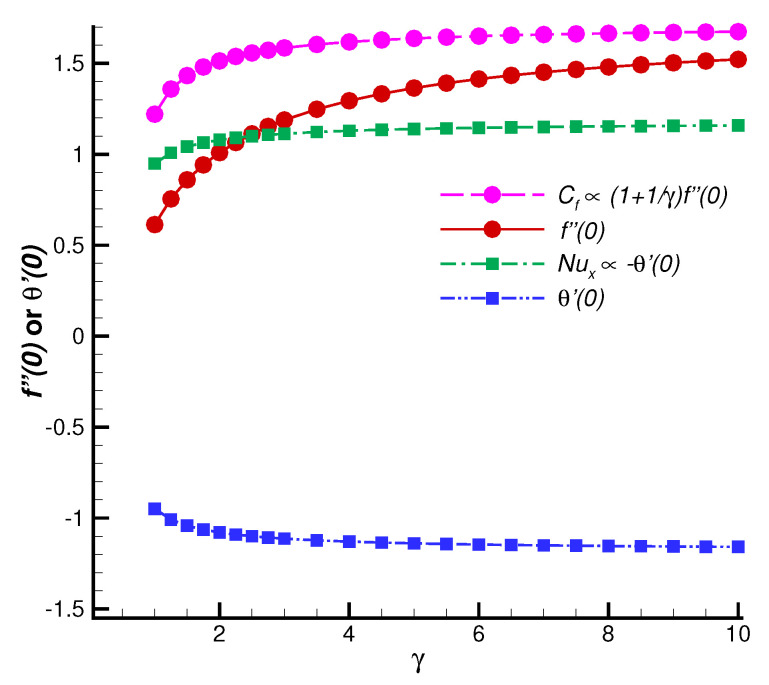
Comparison of convergent series solutions of $f^{\prime \prime }\left(0\right)$ ($C_{f}$) and $\theta^{\prime }\left(0\right)$ ($Nu_{x}$) obtained by HAM with the numerical results for different values of Casson fluid parameter γ when s=2, M=1, Pr=0.71. Line: convergent series solutions; symbols: corresponding numerical results.

To reveal the accuracy and superiority of the series solution, let us first consider the case γ=M=1 with different values of the suction parameter *s*, which has the convergent series solution of $f^{\prime \prime }\left(0\right)$ and $\theta^{\prime }\left(0\right)$. As shown in Figure 2, the convergent values of $f^{\prime \prime }\left(0\right)$ and $\theta^{\prime }\left(0\right)$ obtained by HAM agree quite well with the numerical results [33]. Without the loss of generality, we further investigate the changes of skin friction and Nusselt number with various values of Casson fluid parameter γ, as shown in Figure 3. The convergent values of $f^{\prime \prime }\left(0\right)$ and $\theta^{\prime }\left(0\right)$ are in good agreement with the corresponding numerical results in a region of γ. All of these cases indicate that the analytic formulas of $f^{\prime \prime }\left(0\right)$ and $\theta^{\prime }\left(0\right)$ (55)–(56) are valid, so that one can obtain a sufficiently accurate approximation by means of the optimal convergence-control parameters. Moreover, according to Figure 2, it is seen that both the skin friction and the Nusselt number increase linearly with an increase in *s*. However, the influence of γ is relatively weak, $C_{f}$, $f^{\prime \prime }\left(0\right)$, $Nu_{x}$ and $-\theta^{\prime }\left(0\right)$ increase slowly and nonlinearly with respect to γ.

**Figure 4:**
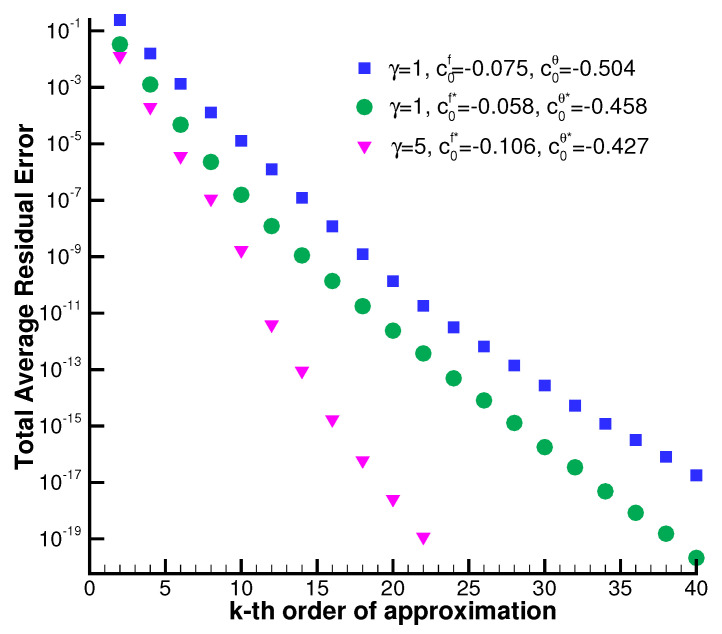
Total average residual error of Equations (8) and (9) in cases of γ=1 and γ=5 for M=3, s=2, Pr=0.71 at different orders of approximation given by HAM using non-optimal $c_{0}^{f}=-0.075$, $c_{0}^{\theta }=-0.504$ (for γ=1), optimal $c_{0}^{f^{*}}=-0.058$, $c_{0}^{\theta^{*}}=-0.458$ (for γ=1) and optimal $c_{0}^{f^{*}}=-0.106$, $c_{0}^{\theta^{*}}=-0.427$ (for γ=5), respectively.

Furthermore, we would like to emphasize two points. It is known that the HAM method bears superiority over other analytical/semi-analytical methods. Firstly, HAM can ensure the convergence accuracy of nonlinear problems, even in those with strong nonlinearity. A comparison of $f^{\prime \prime }\left(0\right)$ and $\theta^{\prime }\left(0\right)$ with the corresponding numerical values for various values of *M*, γ, *s* is illustrated in Table 1. Note that our convergent results are sufficiently accurate with high decimal precision, especially for large values of *M*, γ, *s*. HAM remains independent of small/large physical parameters and provides a convenient way to control the convergence of homotopy series solutions even for large disturbances, which distinguishes it from all other analytic techniques. Secondly, the homotopy approximation quickly converges with the optimal convergence-control parameters chosen. As shown in Figure 4, the total average residual error decreases sharply for each case as the order of approximation increases. Notice that, using the optimal convergence-control parameters $c_{0}^{f^{*}}=-0.058$, $c_{0}^{\theta^{*}}=-0.458$, results in a convergence speed faster than that using non-optimal values, such as $c_{0}^{f}=-0.075$, $c_{0}^{\theta }=-0.504$ for γ=1. The proper convergence-control parameters guarantee the convergence of the homotopy series solution. Furthermore, it is worth noting that other analytic techniques cannot guarantee the same. Thus, this demonstrates an obvious advantage of using HAM. In practice, the sufficiently accurate approximations are obtained in far fewer terms by the optimal convergence-control parameters.

## 5. Discussions

The explicit solutions of the velocity and temperature distribution (46)–(54) are obtained in Section 3, and are valid in the whole domain η≥0. Therefore, according to the explicit solutions, one can investigate the influence of physical quantities, such as Casson fluid parameter γ, magnetic interaction parameter *M*, suction parameter *s* and Prandtl number Pr on the velocity and temperature profiles, skin friction coefficient $C_{f}$ and local Nusselt number $Nu_{x}$.

### 5.1. Effect of γ

**Figure 5:**
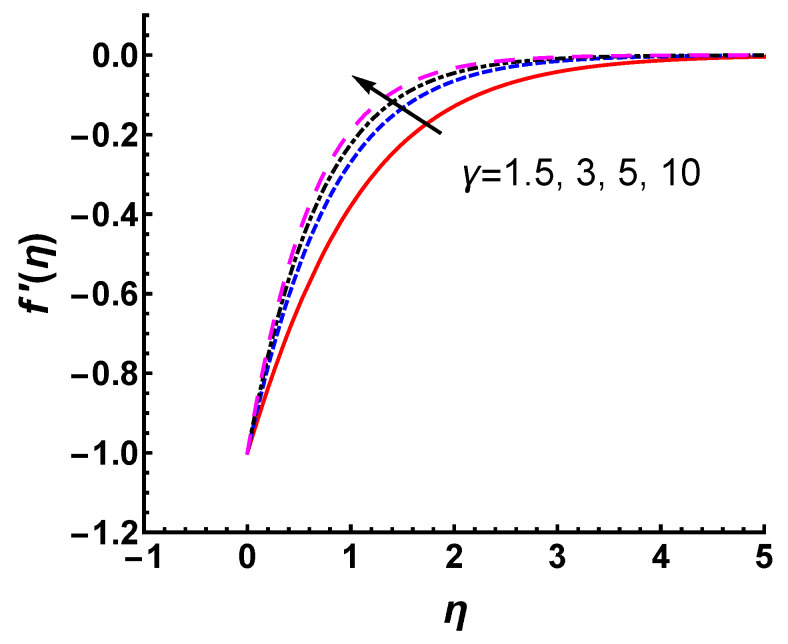
Velocity profile for different values of γ with Pr=0.71, M=1 and s=2.

First, we investigate the effects of Casson fluid parameter γ on the velocity profile and temperature profile. As shown in Figure 5, the magnitude of $f^{\prime }\left(\eta \right)$ decreases as γ increases. The thickness of the velocity boundary layer decreases with increases in γ. This is because the yield stress decreases as γ increases, which results in the velocity being suppressed. It is observed that when γ approaches infinity, the problem in the given case reduces to a Newtonian case. We emphasize that the effect of γ on the velocity profile in this study is consistent with the Casson fluid flow along an exponentially stretching surface [34]. Whether in regard to a stretching or shrinking surface, the magnitude of velocity is found to decrease with increasing γ. The decreasing nature of the momentum boundary layer thickness with increasing γ appears accordingly. This relationship is reasonable in non-Newtonian fluids because an increase in yield stress suppresses the velocity in the boundary layer.

**Figure 6:**
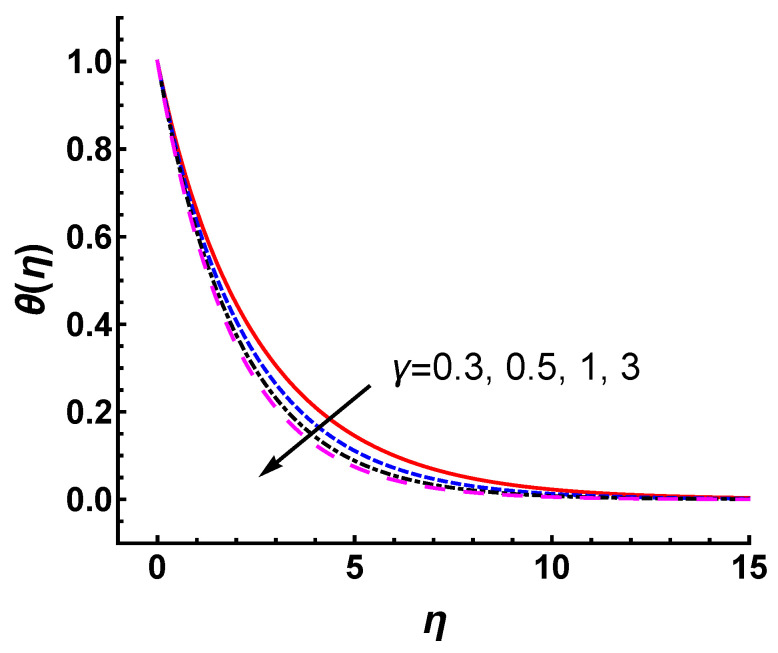
Temperature profile for different values of γ with Pr=0.2, M=2 and s=3.

**Figure 7:**
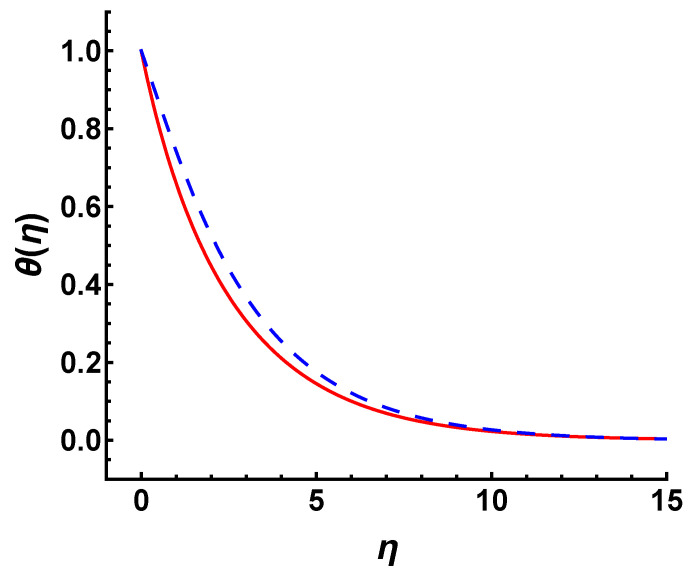
Temperature profile for different wall temperature conditions in the cases of γ=0.3, Pr=0.2, M=2 and s=3. Long-dashed line: $Tw=T_{\infty }+T_{0}e^{\frac{x}{2L}}$; solid line: $Tw=T_{\infty }+T_{0}$.

Figure 6 shows the effect of γ on the temperature profile θ(η) in the cases of M=2, s=3 and Pr=0.2. It can be seen that the temperature decreases slightly with the increasing values of γ. Hence, the thermal boundary layer thickness decreases as the γ increases. Here, we would like to illustrate a point regarding the wall temperature Tw. Note that Tw given by Equation (6) in this study is a uniformly distributed constant. The temperature field given by constant Tw is much more suppressed in the same value of γ than that of the wall temperature condition increasing exponentially with *x* given by $Tw=T_{\infty }+T_{0}e^{\frac{x}{2L}}$. Figure 7 exhibits the temperature profiles for constant Tw and exponentially increased Tw in the cases of γ=0.3, Pr=0.2, M=2 and s=3, respectively. It is observed that the temperature increases for $Tw=T_{\infty }+T_{0}e^{\frac{x}{2L}}$ under the same parameters. Physically, as the wall temperature increases, the temperature of flow within the boundary layer increases. This causes an increase in the thermal boundary layer thickness.

### 5.2. Effects of *M* and *s*

**Figure 8:**
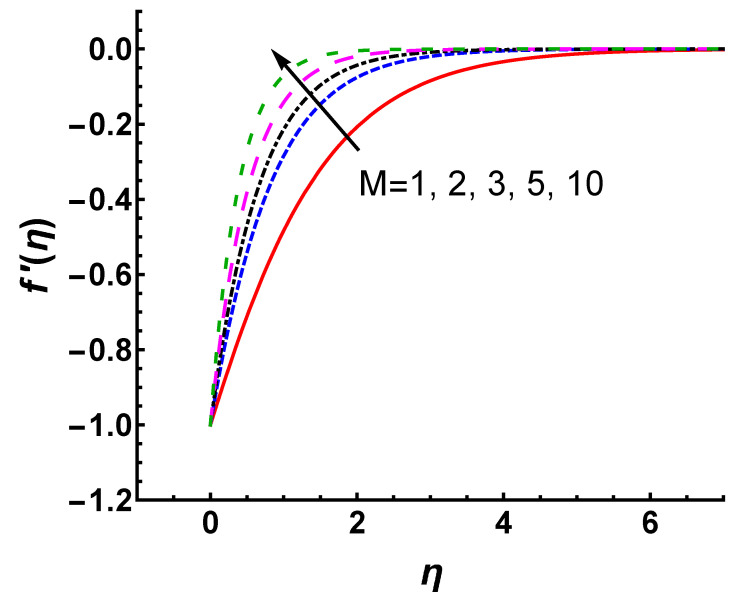
Velocity profile $f^{\prime }\left(\eta \right)$ for different values of *M* with Pr=0.71, γ=1 and s=2.

**Figure 9:**
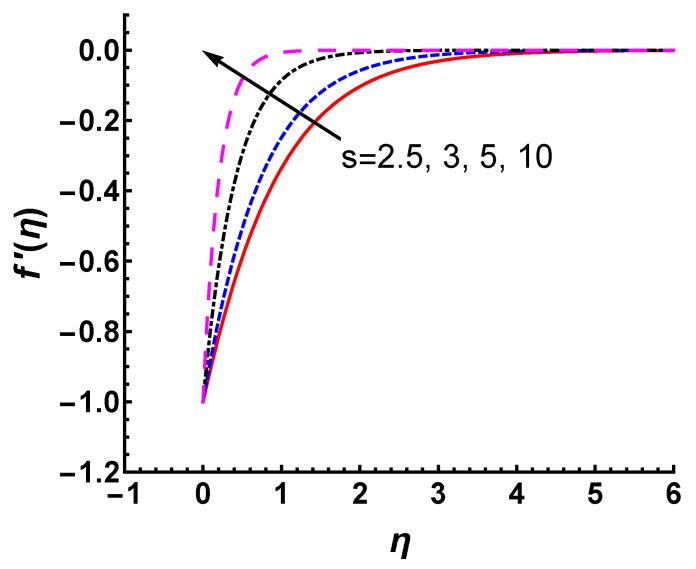
Velocity profile $f^{\prime }\left(\eta \right)$ for different values of *s* with γ=M=1 and Pr=0.71.

The curves of $f^{\prime }\left(\eta \right)$ versus *M* and *s* are shown in Figure 8 and Figure 9, respectively. It is noticed that the magnitude of velocity profiles shows an appreciable decrease for large values of *M* and *s*. From Figure 8, the magnitude of velocity in the boundary layer is suppressed as *M* increases because the force of the magnetic field opposes the motion of the fluid. As shown in Figure 9, the magnitude of velocity decreases significantly with increasing mass suction, which causes a decrease in the boundary layer thickness. This phenomenon can be explained physically. The heated fluid is sucked closer to the wall as the mass suction becomes stronger, where the flow is slowed down due to the greater influence of viscosity. This effect suppresses the maximum velocity in the boundary layer. Therefore, an increased *s* leads to a faster reduction in the magnitude of velocity.

**Figure 10:**
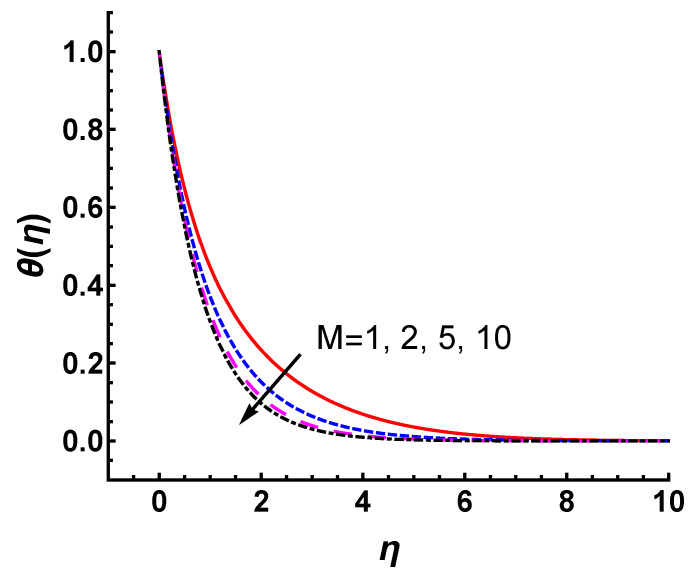
Influence of *M* parameter on temperature profile θ(η) with γ=1, s=2, Pr=0.71; line: HAM results, marker: numerical results.

**Figure 11:**
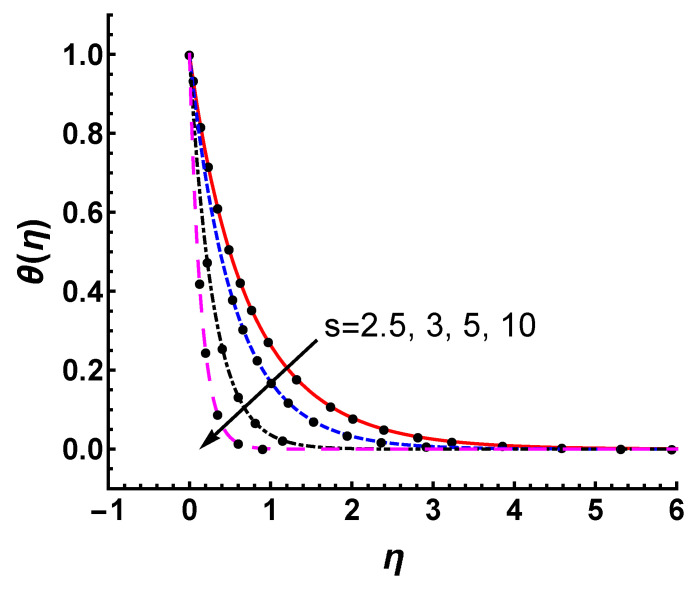
Influence of *s* parameter on temperature profile θ(η) with γ=s=1, Pr=0.71; line: HAM results, marker: numerical results.

Figure 10 and Figure 11 show the effects of *M* and *s* on the temperature profile θ(η). Figure 10 depicts the features of temperature profile as a function of η for various *M*. As *M* increases, the thermal temperature thickness becomes slightly thinner, and is not so sensitive to *M*. The convergent analytic approximation θ(η) for s=2.5, 3, 5 and 10 is shown in Figure 11. The increase in *s* obviously reduces the temperature profile. This change is quite significant due to the increase of mass suction. Compared with *M*, *s* has a greater impact on thermal distribution. Meanwhile, θ(η) obtained by HAM matches the numerical ones quite well for each case, as shown in Figure 11.

*M* and *s* show similar effects on temperature and velocity. Due to the applied magnetic field and suction, the velocity and temperature distributions become more uniform within the boundary layer. The presence of a magnetic field force opposite to the velocity direction and suction tends to reduce the momentum and thermal thickness of the boundary layer. This shows the effect of decreasing both the velocity and temperature within the boundary layer.

**Figure 12:**
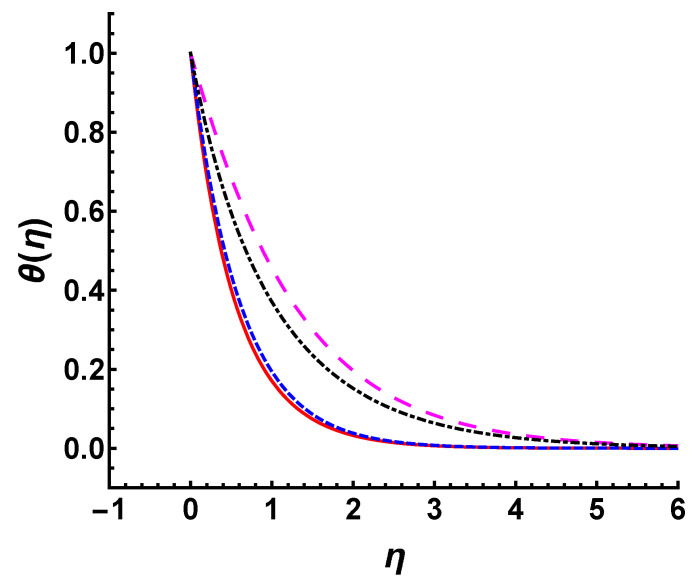
Temperature profile for different wall temperature conditions in the cases of M=2 (γ=1, s=2, Pr=0.71) and s=3 (γ=M=1, Pr=0.71), respectively. Long-dashed line: $Tw=T_{\infty }+T_{0}e^{\frac{x}{2L}}$, M=2; dot-dashed line: $Tw=T_{\infty }+T_{0}$, M=2; short-dashed line: $Tw=T_{\infty }+T_{0}e^{\frac{x}{2L}}$, s=3; solid line: $Tw=T_{\infty }+T_{0}$, s=3.

Additionally, similar to the analysis in γ, we compare the temperature profile for different wall temperature conditions in the cases of M=2 and s=3 (see Figure 12). It is observed that the temperature profile caused by $Tw=T_{\infty }+T_{0}$ decreases faster than that caused by $Tw=T_{\infty }+T_{0}e^{\frac{x}{2L}}$ under the same parameters. The exponentially increasing wall temperature with *x* raises the temperature of the fluid within the boundary layer and increases the thermal boundary layer thickness.

### 5.3. Effect of Pr

**Figure 13:**
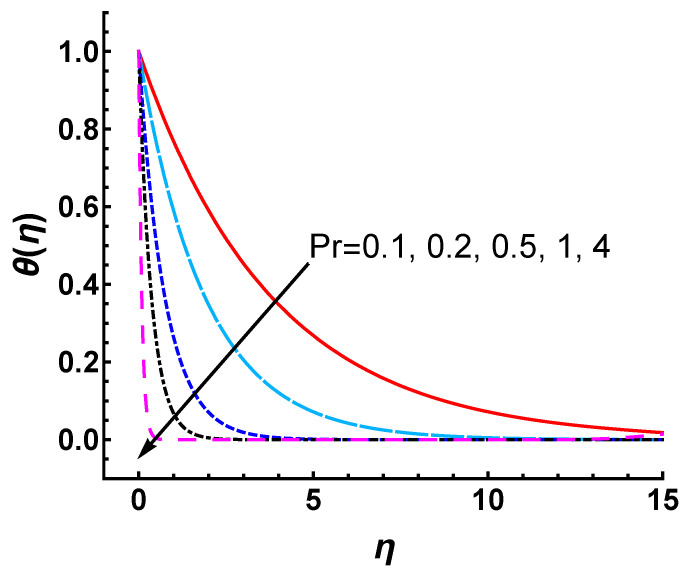
Influence of Pr on temperature profile θ(η) obtained by HAM with M=s=γ=3.

Notice that since the Prandtl number Pr is closely related to the temperature of the boundary layer, we study the influences of Pr on the temperature profile θ(η). As shown in Figure 13, the temperature profile decreases with the increasing values of Pr. The thermal boundary layer thickness is reduced with an increase in Pr. Since Pr signifies the ratio of momentum diffusivity to thermal diffusivity, the thickness of momentum and thermal boundary layers can be controlled using Pr. The heat diffuses faster than the momentum for a small value of Pr. Therefore, the thermal boundary layer is thicker than that of the momentum boundary layer. In other words, higher thermal conductivity corresponds to a thicker thermal boundary layer. Therefore, it is observed from Figure 13 that the heat diffuses slowly and the thermal boundary layer becomes thinner as Pr increases.

### 5.4. Analysis of $C_{f}$ and $Nu_{x}$

The effects of *s* and γ on skin friction $C_{f}$ and Nusselt number $Nu_{x}$ are investigated by the explicit solutions of $f^{\prime \prime }\left(0\right)$ and $\theta^{\prime }\left(0\right)$ in Section 4. Figure 2 illustrates that an increase in *s* leads to a linear increase in $f^{\prime \prime }\left(0\right)$ and linear decrease in $\theta^{\prime }\left(0\right)$. According to Equations (11) and (12), $C_{f}$ and $Nu_{x}$ exhibit a linearly increasing trend as *s* increases. However, $f^{\prime \prime }\left(0\right)$ and $\theta^{\prime }\left(0\right)$ are nonlinear functions of γ (see Figure 3). Note that $C_{f}\propto \left(1+1/\gamma \right)f^{\prime \prime }\left(0\right)$, $C_{f}$ and $Nu_{x}$ increase slowly with an increase in γ. As γ increases to infinity, a Newtonian case appears.

In addition, the comparison of the present results corresponding to $f^{\prime \prime }\left(0\right)$ and $\theta^{\prime }\left(0\right)$ with numerical solutions for various values of *M*, γ and *s* is presented in Table 1. It shows the high accuracy of the applied scheme and verifies the effectiveness of the HAM approach. From Table 1, it is seen that the influence of *M* on $f^{\prime \prime }\left(0\right)$ and $\theta^{\prime }\left(0\right)$ is similar to γ. An increment in *M* causes $f^{\prime \prime }\left(0\right)$ and $-\theta^{\prime }\left(0\right)$ to increase slowly. The same is true for the trend of $C_{f}$ and $Nu_{x}$ due to Equations (11) and (12).

**Figure 14:**
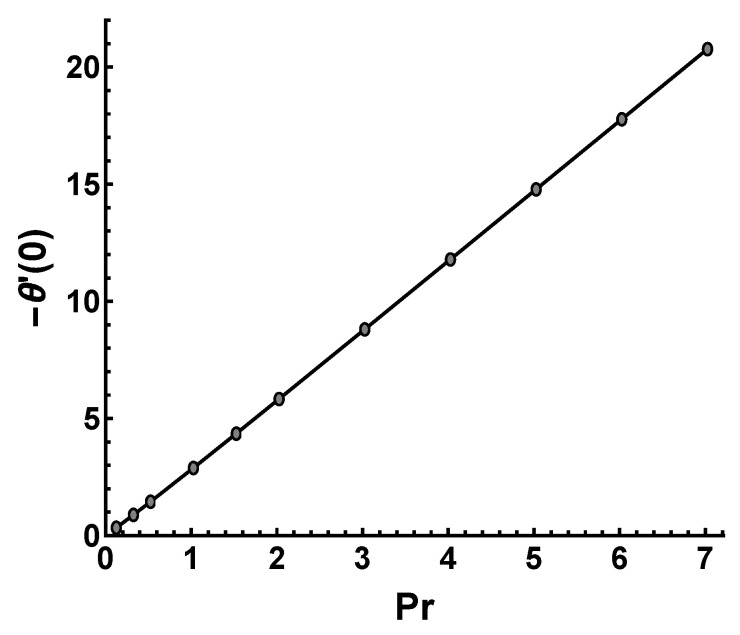
Influence of Pr on $-\theta^{\prime }\left(0\right)$ obtained by HAM with M=s=γ=3.

Figure 14 displays the wall temperature gradient $-\theta^{\prime }\left(0\right)$ against Pr. It is clearly shown that the increment in Pr leads to a linear increase in the wall temperature gradient. The wall temperature gradient is proportional to the heat transfer rate or $Nu_{x}$. As mentioned earlier, Pr represents the ratio of momentum diffusivity to thermal diffusivity. The momentum diffusivity increases, whereas thermal diffusivity decreases as Pr increases, so the heat transfer rate increases. This reflects that the Nusselt number $Nu_{x}$ increases with the increasing values of Pr. Significantly, the heat transfer rate under the condition of an exponentially increasing wall temperature is lower than that under a constant wall temperature. As can be observed from Table 2, the convergent solution $-\theta^{\prime }\left(0\right)$ is reduced by increasing the wall temperature along the *x*-direction. This means that the temperature profile within the boundary layer enhances, which causes an increase in thermal boundary layer thickness.

Finally, it should be emphasized that our series solutions are sufficiently accurate by comparison with the numerical results. In addition, as observed in Figure 4, the squared residual error of the homotopy approximations decreases exponentially as the order of approximation increases. Thus, it is in fact unnecessary to compare our convergent results with numerical ones. The variation of the squared residual error evaluates the accuracy of the homotopy approximation.

## 6. Conclusions

The MHD flow and heat transfer of a Casson fluid over an exponentially shrinking sheet with suction is investigated using the HAM approach. First, the governing boundary layer equations are transformed into nonlinear ordinary differential equations using similarity transformations. Then, the nonlinear ordinary differential governing Equations (8) and (9) are replaced utilizing an infinite number of linear sub-equations, which are solved analytically in the whole domain. Due to the freedom in constructing the zeroth-order deformation equations, we can choose the appropriate initial guesses and the auxiliary linear operators so that the explicit solutions are derived further. The generosity in freedom is based on the concept of convergence-control by means of convergence-control parameters. The optimal values of the convergence-control parameters are strongly suggested for use in practice. In general, it is enough to obtain an accurate homotopy approximation by using optimal convergence-control parameters determined by the minimum of the squared residual error corresponding to the governing equations.

In this study, by solving the coupled nonlinear differential equations in the MHD flow and heat transfer of a Casson fluid over an exponentially shrinking sheet, we arrive at the following main points:

- The explicit analytic solutions of f(η) and θ(η) are obtained and valid in the whole region η=[0,+∞).
- The important quantities $f^{\prime \prime }\left(0\right)$ and $\theta^{\prime }\left(0\right)$ related to the skin friction coefficient $C_{f}$ and local Nusselt number $Nu_{x}$ are derived in an explicit form.
- The convergent analytic solutions are in good agreement with the numerical solutions. The rapid decrease in squared residual error ensures the accuracy of the homotopy approximation.
- An increase in the Casson fluid parameter γ suppresses the magnitude of velocity profile $f^{\prime }\left(\eta \right)$ due to the reduced yield stress as γ increases. This leads to a thinner momentum boundary layer thickness. The velocity profile magnitude $f^{\prime }\left(\eta \right)$ is found to decrease with increasing γ for both stretching and shrinking surfaces.
- The temperature profile θ(η) decreases slightly with increasing values of γ in the current case, which decreases the thermal boundary layer thickness.
- The magnitudes of $f^{\prime }\left(\eta \right)$ and θ(η) decrease significantly with increases in the magnetic interaction parameter *M* and suction parameter *s*.
- The velocity and thermal boundary layer thicknesses decrease as *M* and *s* increase. The presence of a magnetic field force opposite to the velocity and suction reduces the momentum and thermal thickness of the boundary layer.
- The temperature profile and thermal boundary layer thickness decrease with increasing values of Prandtl number Pr. The heat diffuses faster corresponding to the higher thermal conductivity for a small value of Pr.
- $C_{f}$ and $Nu_{x}$ exhibit a linearly increasing trend as *s* becomes stronger.
- $C_{f}$ and $Nu_{x}$ increase nonlinearly with increases in γ and *M*.
- The wall heat transfer rate $-\theta^{\prime }\left(0\right)$ increases linearly as Pr increases, as a result, $Nu_{x}$ also increases linearly.
- Compared with the constant wall temperature condition, the exponentially increasing wall temperature with *x* raises the temperature of the fluid within the boundary layer and leads to increased thickness of the thermal boundary layer.

## Figures and Tables

**Table 1:** Analytic approximations (HAM) of $f^{\prime \prime }\left(0\right)$, $\theta^{\prime }\left(0\right)$ compared with numerical results for various *M*, γ and *s* in case of Pr=0.71.

Parameters		$f^{\prime \prime }\left(0\right)\;$		$\theta^{\prime }\left(0\right)\;$	
		HAM	Num	HAM	Num
*M*	2	1.18455279	1.184553	−1.10536254	−1.105533
	3	1.47878566	1.478786	−1.14720967	−1.147279
	5	1.89590282	1.895903	−1.18877394	−1.188803
	10	2.60700267	2.607003	−1.23492995	−1.234942
γ	1	0.61356181	0.613026	−0.94909557	−0.944217
	3	1.18865714	1.188657	−1.11302087	−1.113159
	5	1.36433170	1.364332	−1.13918925	−1.139268
	10	1.52194504	1.521945	−1.15851866	−1.158571
*s*	2.5	1.00391561	1.003915	−1.48397701	−1.483987
	3	1.31012112	1.310121	−1.90669518	−1.906695
	5	2.39570505	2.395705	−3.42764833	−3.427648
	10	4.94949146	4.949491	−7.04073641	−7.040736

**Table 2:** Analytic approximations (HAM) of $-\theta^{\prime }\left(0\right)$ under different wall temperature conditions for various Pr with M=γ=s=3.

Parameters		$-\theta^{\prime }\left(0\right)\;$	
		$Tw=T_{\infty }+T_{0}$	$Tw=T_{\infty }+T_{0}e^{\frac{x}{2L}}$
Pr	0.1	0.2664	0.23258
	0.2	0.53845	0.47562
	0.5	1.37692	1.24919
	1	2.81671	2.62471
	4	11.71837	11.42673

## Data Availability

The data presented are available in this article.

## Appendix A. The Related Coefficients in $b_{m,n}^{k}$

The related coefficients appearing in $b_{m,n}^{k}$ of Equations (37)–(45) are given below.

(1) $\Gamma_{m,n}^{q}$ is defined by

(A1) $$\begin{array}{cc} \; & \Gamma_{m,1}^{q}=c_{0}^{f}\left(\left(1+\frac{1}{\gamma }\right)\lambda^{2}{\hat{d}}_{m-1,1}^{q}-M{\hat{c}}_{m-1,1}^{q}+\lambda \delta_{m,1}^{q}\right),\;0\leq q\leq m-1, \end{array}$$

(A2) $$\begin{array}{cc} \; & \Gamma_{m,m+1}^{0}=c_{0}^{f}\left(\lambda \delta_{m,m+1}^{0}-2\lambda {\hat{\delta }}_{m,m+1}^{0}\right), \end{array}$$

(A3) $$\begin{array}{cc} \; & \Gamma_{m,n}^{q}=c_{0}^{f}\left(\left(1+\frac{1}{\gamma }\right)\lambda^{2}{\hat{d}}_{m-1,n}^{q}-M{\hat{c}}_{m-1,n}^{q}+\lambda \delta_{m,n}^{q}-2\lambda {\hat{\delta }}_{m,n}^{q}\right),\;0\leq q\leq m-n, \end{array}$$

(A4) $$\begin{array}{cc} \; & \Gamma_{m,n}^{q}=c_{0}^{f}\left(\lambda \delta_{m,n}^{q}-2\lambda {\hat{\delta }}_{m,n}^{q}\right),\;q=m-n+1, \end{array}$$

(A5) $$\begin{array}{cc} \; & \Gamma_{m,n}^{q}=0,\;\mathrm{otherwise}, \end{array}$$

where

(A6) $$\begin{array}{cc} \; & {\hat{d}}_{m,m+1}^{0}=-{\left(m+1\right)}^{3}b_{m,m+1}^{0}, \end{array}$$

(A7) $$\begin{array}{cc} \; & {\hat{d}}_{m,k}^{i}=\left(i+1\right)c_{m,k}^{i+1}-kc_{m,k}^{i},\;0\leq i\leq m-k,\;1\leq k\leq m, \end{array}$$

(A8) $$\begin{array}{cc} \; & {\hat{d}}_{m,k}^{i}=-kc_{m,k}^{i},\;i=m-k+1,\;1\leq k\leq m, \end{array}$$

in which

(A9) $$\begin{array}{cc} \; & c_{m,m+1}^{0}={\left(m+1\right)}^{2}b_{m,m+1}^{0}, \end{array}$$

(A10) $$\begin{array}{cc} \; & c_{m,k}^{i}=\left(i+1\right)\left(i+2\right)b_{m,k}^{i+2}-2k\left(i+1\right)b_{m,k}^{i+1}+k^{2}b_{m,k}^{i},\;0\leq i\leq m-k-1,\;1\leq k\leq m, \end{array}$$

(A11) $$\begin{array}{cc} \; & c_{m,k}^{i}=-2k\left(i+1\right)b_{m,k}^{i+1}+k^{2}b_{m,k}^{i},\;i=m-k,\;1\leq k\leq m, \end{array}$$

(A12) $$\begin{array}{cc} \; & c_{m,k}^{i}=k^{2}b_{m,k}^{i},\;i=m-k+1,\;1\leq k\leq m, \end{array}$$

and for 1≤n≤m+1, 0≤q≤m−n+1,

(A13) $$\begin{array}{cc} \; & \delta_{m,n}^{q}=\sum_{k=0}^{m-1}\sum_{j=J_{0}}^{J_{1}}\sum_{i=I_{0}}^{I_{1}}c_{k,j}^{i}\;\beta_{m-1-k,n-j}^{q-i}b_{m-1-k,n-j}^{q-i},\\ \; & J_{0}=max\left\{1,n+k-m\right\},\\ \; & J_{1}=min\left\{n,k+1\right\},\\ \; & I_{0}=max\left\{0,q-\left(m-k-n+j\right)\right\},\\ \; & I_{1}=min\left\{q,k-j+1\right\}, \end{array}$$

in which

(A14) $$\beta_{m,k}^{i}=\left\{\begin{array}{cc} 0, & \;m=k=0,\;i\geq 1,\\ 0, & \;m>0,\;k=0,\;i\geq 1,\\ 0, & \;k>m+1,\\ 0, & \;i>m-k+1\\ 1, & \;\mathrm{otherwise}, \end{array}\right.$$

and

(A15) $$\begin{array}{cc} \; & {\hat{c}}_{m,m+1}^{0}=-\left(m+1\right)b_{m,m+1}^{0}, \end{array}$$

(A16) $$\begin{array}{cc} \; & {\hat{c}}_{m,k}^{i}=\left(i+1\right)b_{m,k}^{i+1}-kb_{m,k}^{i},\;0\leq i\leq m-k,\;1\leq k\leq m, \end{array}$$

(A17) $$\begin{array}{cc} \; & {\hat{c}}_{m,k}^{i}=-kb_{m,k}^{i},\;i=m-k+1,\;1\leq k\leq m, \end{array}$$

and

(A18) $$\begin{array}{cc} \; & {\hat{\delta }}_{m,n}^{q}=\sum_{k=0}^{m-1}\sum_{j=J_{0}}^{J_{2}}\sum_{i=I_{0}}^{I_{1}}{\hat{c}}_{k,j}^{i}\;{\hat{c}}_{m-1-k,n-j}^{q-i},\;2\leq n\leq m+1,\;0\leq q\leq m-n+1, \end{array}$$

where $J_{0}$, $I_{0}$, $I_{1}$ are shown in Equations (A13), $J_{2}=min\left\{n-1,k+1\right\}$.

(2) $\mu_{n,k}^{q}$ in Equations (37)–(45) is defined by

(A19) $$\begin{array}{cc} \; & \mu_{1,k}^{q}=\frac{q!\;(2-k+q)}{k!},\;0\leq k\leq q+1,\;q\geq 0, \end{array}$$

(A20) $$\begin{array}{cc} \; & \mu_{n,k}^{q}=\frac{q!}{k!}\sum_{j=k}^{q}\frac{\left(j-k+1\right)}{{\left(n-1\right)}^{q-j+1}}\frac{1}{n^{j-k+2}},\;0\leq k\leq q,\;n\geq 2,\;q\geq 0. \end{array}$$

## Appendix B. The Related Coefficients in $d_{m,n}^{l}$

The related coefficients appearing in $d_{m,n}^{l}$ of Equations (47)–(53) are as follows.

(1) $\Omega_{m,n}^{q}$ in Equations (47)–(53) is defined by

(A21) $$\begin{array}{cc} \; & \Omega_{m,n}^{q}=c_{0}^{\theta }\left(\lambda \varpi_{m-1,n}^{q}+Pr\kappa_{m,n}^{q}\right),\;1\leq n\leq m,\;0\leq q\leq m-n, \end{array}$$

(A22) $$\begin{array}{cc} \; & \Omega_{m,n}^{q}=c_{0}^{\theta }Pr\kappa_{m,n}^{q},\;2\leq n\leq m+1,\;q=m-n+1, \end{array}$$

in which

(A23) $$\begin{array}{cc} \; & \varpi_{m,m+1}^{0}={\left(m+1\right)}^{2}d_{m,m+1}^{0}, \end{array}$$

(A24) $$\begin{array}{cc} \; & \varpi_{m,k}^{i}=\left(i+2\right)\left(i+1\right)d_{m,k}^{i+2}-2k\left(i+1\right)d_{m,k}^{i+1}+k^{2}d_{m,k}^{i},\;1\leq k\leq m,\;0\leq i\leq m-k-1, \end{array}$$

(A25) $$\begin{array}{cc} \; & \varpi_{m,k}^{m-k}=-2k\left(m-k+1\right)d_{m,k}^{m-k+1}+k^{2}d_{m,k}^{m-k},\;1\leq k\leq m, \end{array}$$

(A26) $$\begin{array}{cc} \; & \varpi_{m,k}^{m-k+1}=k^{2}d_{m,k}^{m-k+1},\;1\leq k\leq m, \end{array}$$

and

(A27) $$\begin{array}{cc} \; & \kappa_{m,n}^{q}=\sum_{k=0}^{m-1}\sum_{j=J_{0}^{*}}^{J_{1}^{*}}\sum_{i=I_{0}^{*}}^{I_{1}^{*}}\tau_{k,j}^{i}\;\beta_{m-1-k,n-j}^{q-i}\;b_{m-1-k,n-j}^{q-i},\\ \; & 1\leq n\leq m+1,\;0\leq q\leq m+1-n,\\ \; & J_{0}^{*}=max\left\{1,n+k-m\right\},\\ \; & J_{1}^{*}=min\left\{n,k+1\right\},\\ \; & I_{0}^{*}=max\left\{0,q-\left(m-k-n+j\right)\right\},\\ \; & I_{1}^{*}=min\left\{q,k-j+1\right\}, \end{array}$$

where

(A28) $$\begin{array}{cc} \; & \tau_{m,m+1}^{0}=-\left(m+1\right)d_{m,m+1}^{0}, \end{array}$$

(A29) $$\begin{array}{cc} \; & \tau_{m,k}^{i}=\left(i+1\right)d_{m,k}^{i+1}-kd_{m,k}^{i},\;1\leq k\leq m,\;0\leq i\leq m-k, \end{array}$$

(A30) $$\begin{array}{cc} \; & \tau_{m,k}^{m-k+1}=-kd_{m,k}^{m-k+1},\;1\leq k\leq m, \end{array}$$

note $\beta_{m,k}^{i}$ is defined by Equation (A14).

(2) $w_{n,i}^{q}$ in Equations (47)–(53) is defined by

(A31) $$\begin{array}{cc} \; & w_{1,i}^{q}=-\frac{q!}{i!},\;0\leq i\leq q+1, \end{array}$$

(A32) $$\begin{array}{cc} \; & w_{n,i}^{q}=\frac{q!}{i!}\sum_{j=i}^{q}\frac{q!}{i!}\frac{1}{{\left(n-1\right)}^{q-j+1}}\frac{1}{n^{j-i+1}},\;n\geq 2,\;0\leq i\leq q, \end{array}$$

for q≥0.
